# Dental Management of a Patient with Special Health Care Needs

**DOI:** 10.1155/2021/6692325

**Published:** 2021-02-03

**Authors:** Leopoldo Lucio da Mata, Ana Maria Schroden Rodrigues da Cunha, Andrezza Morais Moronte

**Affiliations:** ^1^Mário Palmério University Hospital, University of Uberaba, Uberaba, Minas Gerais, Brazil; ^2^Department of Pediatric Dentistry at University of Uberaba, Uberaba, Minas Gerais, Brazil

## Abstract

Patients with special health care needs (PSHCN) may have an increased risk of oral disease throughout the course of their life and require particular delivery of dental care due to their medical condition or limitations. The purpose is to report the dental management of a patient with Cornelia de Lange Syndrome (CdLS), which was classified as PSHCN due to physical, behavioural, cognitive, and emotional impairment. A 14-year-old female with a clinical diagnosis of CdLS and its common craniofacial features such as microcephaly, short neck, synophrys, arched eyebrows, downturned angle of the mouth, high arched palate, micrognathia, and microdontia was referred to the hospital where the dental treatment was performed under general anesthesia. Multiple tooth extraction, fillings, and coronary polishing were performed. During the follow-up, we observed that dental restorations were clinically satisfactory and there was an improvement in the patient's behaviour during dental treatment. Managing and shaping behaviour of such patients are crucial to delivering quality dental care, as they require specialized care due to their behavioural and clinical conditions.

## 1. Introduction

Patients with special health care needs (PSHCN) may have an increased risk of oral disease throughout the course of life [1] and require particular delivery of dental care due to their medical condition and limitations [1, 2]. These patients also need additional supports to maintain their oral health and access oral health care services [3]. Oral diseases may have a direct and critical impact on their health and quality of life [1].

Cornelia de Lange Syndrome (CdLS) is a rare syndrome of multiple congenital anomalies with unknown aetiology, characterised by distinct clinical characteristics such as facial appearance, prenatal and postnatal growth deficiency, feeding difficulties, psychomotor delays and problems, behavioural problems, and anomalies of the extremities. The diagnosis of this syndrome is mainly clinical [4–6]. The most common craniofacial features are microcephaly, short neck with low anterior and posterior hairlines, synophrys, long curly eyelashes, downturned angles of the mouth, high arched palate, micrognathia, macroglossia, microdontia, delayed tooth eruption, and partial anodontia [5, 7–9].

The purpose of this case report is to endeavor further guidance for dental practitioners relating to the dental management of patients with CdLS which is classified as PSHCN due to physical, behavioural, cognitive, and emotional impairment.

## 2. Case Report

A 14-year-old female, brown skinned, had a clinical diagnosis of CdLS. She had common craniofacial features for CdLS such as microcephaly, short neck, synophrys, arched eyebrows, long curly eyelashes, downturned angle of the mouth, high arched palate, micrognathia, microdontia, delayed tooth eruption, and partial anodontia. The patient's mother sought the dental service at the Basic Health Unit (UBS) in Sacramento, Brazil. However, there were no specialized dentists that treat PSHCN. Therefore, the patient was referred to our Pediatric Dental Clinic at University of Uberaba, Uberaba, Brazil. In the first appointment, we tried to talk and persuaded the patient to open her mouth, although we did not succeed due to her aggressive behaviour. After the first attempt, we proposed to use physical restraints to undertake the treatment, though her mother did not agree with it. Owing to the patient's behaviour, we were not able to conduct any intraoral examination as well as radiographic examination. Thus, we referred her to the Mário Palmério University Hospital, Uberaba, Brazil, for dental treatment under general anesthesia. Firstly, she underwent preanesthetic assessment, such as complete blood test and chest X-ray. Thus, she had an appointment with an anaesthesiologist who authorised her for the dental treatment under general anesthesia.

The patient was sedated and orally intubated by the anaesthesiologist assisted by a nurse anesthetist. A dose of prophylactic antibiotic of cefazolin sodium (1 g), anti-inflammatory dexamethasone (0.1 mg/kg body weight), and analgesic paracetamol (10 mg/kg body weight) was administered intravenously. Afterwards, the skin asepsis of the patient's face was realised with 2% aqueous solution of chlorhexidine digluconate and sterile surgical drapes were placed on her face. We were then able to undertake intraoral examination and carry out a dental treatment plan.

**Figure 1:**
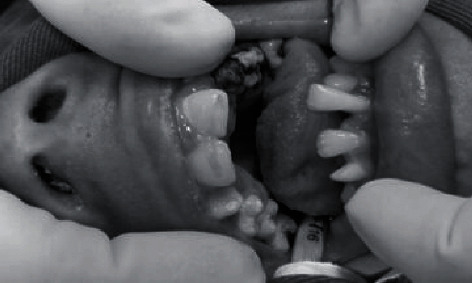
The patient's oral cavity before the treatment.

We observed that the permanent mandibular right second molar, the primary maxillary left first molar, and second molar presented carious lesions with pulp involvement. The permanent maxillary right first molar, the permanent maxillary right second premolar, and the permanent maxillary right and left central incisor had carious lesions extending into the dentin. Furthermore, the permanent mandibular right central incisor was overerupted and injured the patient's palate (Figure 1).

**Figure 2:**
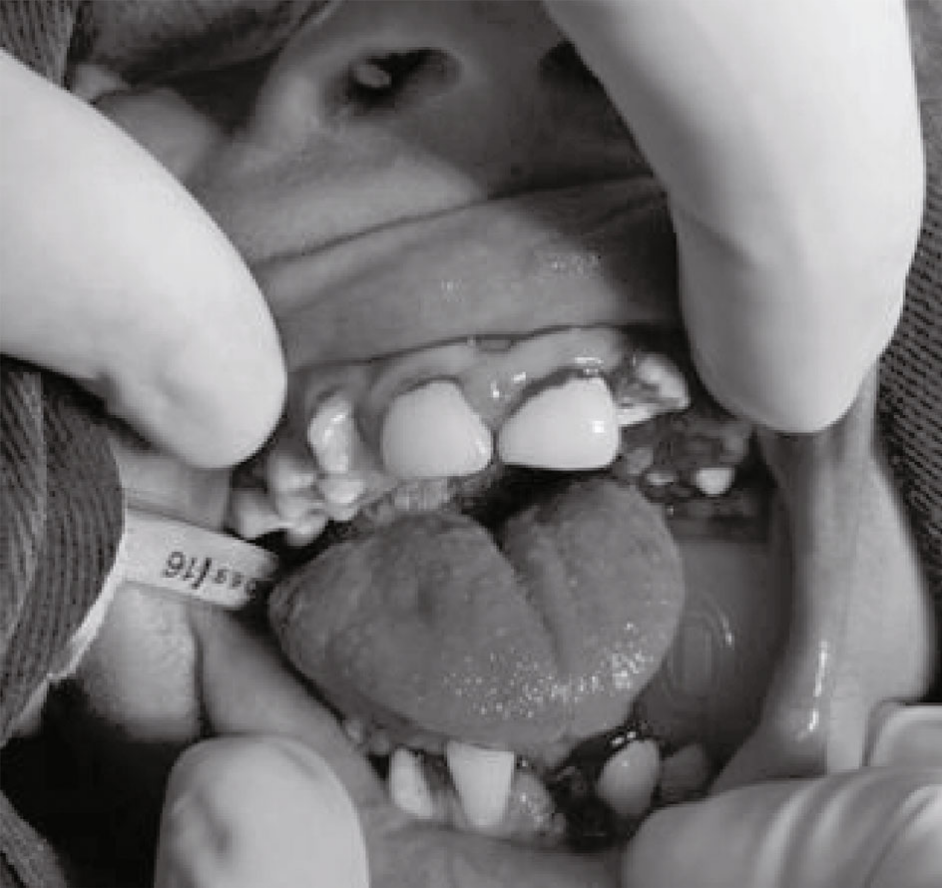
The patient's oral cavity after the dental procedure.

Firstly, dental prophylaxis with prophylaxis paste, pumice stone, and nylon-bristle brush (Prophy brush) was performed to remove the dental biofilm. Secondly, the teeth with pulp involvement and the overerupted tooth were extracted. Afterwards, the teeth with carious extending into the dentin were filled with resin composite (Figure 2).

Postoperatively, the patient was transferred to the postanesthesia care unit where she was closely monitored and assessed for any deterioration in her condition. The patient was discharged on the same day, and paracetamol syrup (10 mg/kg body weight) was prescribed in case of any pain. The follow-up was 1 week and 6 months after, when dental prophylaxis and topical application of acidulated phosphate fluoride gels (APF, 1.23%) were performed. During intraoral examination, we observed that dental restorations were clinically satisfactory and an improvement in her oral health.

Initially, the management of the patient was difficult due to her aggressive behaviour; however, after the procedure was performed under general anesthesia, we observed an improvement in the patient's subsequent behaviour during dental care. Furthermore, this improvement was also reported by her mother. There were no complications during the proposed dental treatment. Unfortunately, we could not keep following the patient due to difficulties to pay for travel from her town to our dental clinic. Therefore, we referred her back to the dental service at the Basic Health Unit (UBS) in her town.

## 3. Discussion

The patient's mother sought a dentist at the UBS in her city; however, it was not possible to perform the dental treatment, as there was not a qualified dentist to provide dental care to patients with special health care needs. The dentists must have specialized knowledge and skills to attend to PSHCN, as these patients may have medical conditions that require extraordinary dental care [10]. Providing access to dental care services to these patients is essential to maintain adequate oral health as they have an increased risk of oral diseases throughout their life, and these oral diseases may directly affect their quality of life and general health [1].

Dentists should consider the patient's behaviour, because it may complicate the delivery of dental care. They should attempt verbal conditioning for permission to undertake the dental treatment, trying to gain the patient's cooperation in the least restrictive manner [11, 12]. If this fails, the dentists should consider using physical and/or chemical restraints. However, before using any restraints, the dentists must obtain consent from the patient's legal guardian and choose the least restrictive technique that will allow the dentist to provide dental care safely [11, 13]. The last option is to refer the patient to undertake dental treatment under general anesthesia [11, 12]. In this case report, after the first unsuccessful attempt at verbal conditioning, the mother did not grant permission for the use of physical restraint. However, it was not possible to perform sedation in the outpatient setting, due to the complexity of the treatment to be performed. Therefore, the dental treatment was performed under general anesthesia, thus achieving the successful accomplishment of the proposed dental treatment.

## 4. Strength and Limitation

The strength of our case report was that our dental clinic works together with the university hospital to provide free multidisciplinary care to all our patients with special health care needs. Some limitations should be nevertheless considered. Firstly, we could not perform dental X-rays and we lacked clinical information about tooth development and tooth anomalies and if there were any cysts or tumours in jaw bones. Furthermore, we were not able to follow-up the patient for more than 6 months due to her family's financial situation.

## 5. Conclusion

Patients with special health care needs require a specialized approach and care owing to their health condition and behaviour. Managing and shaping behaviour of such patients are crucial to delivering high standard dental care. These patients are at higher risk of oral and dental problems and also have lower levels of hygiene practice, such as toothbrushing and dental flossing. Therefore, dental professionals play a crucial role in providing dental care and in maintaining a good level of oral health in these patients.

## Data Availability

No data were used to support this study.

## References

[1] Anders P. L., Davis E. L. (2010). Oral health of patients with intellectual disabilities: a systematic review. *Special Care in Dentistry*.

[2] Newacheck P. W., McManus M., Fox H. B., Hung Y. Y., Halfon N. (2000). Access to health care for children with special health care needs. *Pediatrics*.

[3] Espinoza K. M., Heaton L. J. (2016). Communicating with patients with special health care needs. *Dental Clinics of North America*.

[4] Filippi G. (1989). The de Lange syndrome. Report of 15 cases. *Clinical Genetics*.

[5] Huang W. H., Porto M. (2002). Abnormal first-trimester fetal nuchal translucency and Cornelia de Lange syndrome. *Obstetrics and Gynecology*.

[6] Kline A. D., Grados M., Sponseller P. (2007). Natural history of aging in Cornelia de Lange syndrome. *American Journal of Medical Genetics Part C: Seminars in Medical Genetics*.

[7] Badoe E. (2006). Classical Cornelia de Lange syndrome. *Ghana Medical Journal*.

[8] Braddock S. R., Lachman R. S., Stoppenhagen C. C. (1993). Radiological features in Brachmann-de Lange syndrome. *American Journal of Medical Genetics*.

[9] Jackson L., Kline A. D., Barr M. A., Koch S. (1993). de Lange syndrome: a clinical review of 310 individuals. *American Journal of Medical Genetics*.

[10] (2006). *Special Care in Oral Health*.

[11] Garcia M. J. N., Martinez M. R. M., Sanjuán C. M., Gallardo López N. E., Carracedo Cabaleiro E., Alonso García Y. (2007). Program for coordinated care under general anesthesia for children with special needs. *Medicina Oral, Patología Oral y Cirugía Bucal*.

[12] American Academy of Pediatric Dentistry (2016). Guideline on behavior guidance for the pediatric dental patient. *Pediatric Dentistry*.

[13] National Institute of Dental and Craniofacial Research (2009). *Practical Oral Care for People with Developmental Disabilities*.

